# Hereditary angioedema C1-esterase inhibitor replacement therapy and coexisting autoimmune disorders: findings from a claims database

**DOI:** 10.1186/s13223-020-00439-9

**Published:** 2020-05-27

**Authors:** Henriette Farkas, Donald Levy, Dylan Supina, Melvin Berger, Subhransu Prusty, Moshe Fridman

**Affiliations:** 1 Hungarian Angioedema Reference Center, 3rd Department of Internal Medicine, Semmelweis University, Kutvolgyi ut 4, H-1125 Budapest, Hungary; 2 Allergy & Immunology Services, University of California, 705 W La Veta Ave, Orange, CA USA; 3 CSL Behring, 1020 1st Ave, King of Prussia, PA USA; 4 CSL Behring GmbH, Emil-von-Behring-Straße 76, Marburg, Germany; 5 AMF Consulting Inc., Los Angeles, CA USA

**Keywords:** Plasma-derived C1-esterase inhibitor, Hereditary angioedema, Autoimmune disorders, Claims database, Lupus erythematosus

## Abstract

In this letter to the editor, we present results of claims data analysis. This claims data analysis supports a hypothesis that in patients with hereditary angioedema due to C1-esterase inhibitor (C1-INH) deficiency, the occurrence and/or symptomatology of coexisting autoimmune disease may be positively influenced by a replacement therapy with plasma derived C1-INH.

## To the editor

In hereditary angioedema (HAE), a mutation in the SERPING1 gene causes either deficiency or dysfunctional C1-esterase inhibitor (C1-INH), resulting in activation of contact-kinin system and increased production of bradykinin leading to episodic angioedema [1]. Deficiency of C1-INH leads to enhanced consumption of C2 and C4, which may predispose for autoimmune disease (AD) [2]. Plasma-derived C1-INH (pdC1-INH) is safe and effective for acute treatment and prevention of HAE attacks [3].

To explore the potential association between the type of treatment for HAE due to C1-INH deficiency (C1-INH-HAE) and ADs, we compared coexisting ADs claims frequencies in C1-INH-HAE patients treated with pdC1-INH versus “Other (non-C1-INH)” treatments.

C1-INH-HAE patients were identified in the IMS Health *PharMetrics Plus* claims database for the period January 2012 to December 2016 by International Classification of Diseases (ICD) 9/10 diagnosis code, and classified into “pdC1-INH” (Cinryze and Berinert) or “Other (non-C1-INH) treatment” (Firazyr and Kalbitor) groups.

Patients with at least 1 diagnosis code for C1-INH-HAE including ICD-9-CM 277.6 and 277.8 or ICD-10-D84.1 were included. Patients were required to have an initial Cinryze, Berinert, Firazyr, or Kalbitor pharmacy or medical claim for HAE (index date) from 01 January 2012 to 31 December 2015 (the identification period) and to be continuously enrolled in the same health plan for at least 12 months (the follow-up period) through 31 December 2016. For patients using pdC1-INH, the first fill was used as the index date even if other HAE medications had been used previously. Frequency of visit claims for AD was identified by diagnostic codes.

The association between HAE treatment and AD visit frequency was summarized by overall patients and by gender and age group. The prevalence of patients with at least 1 visit for an AD considered in the study were reported by treatment group. Mean (95% confidence interval [CI]), standard deviation (SD), and range for the visits per patient per year (PPPY) by treatment group, stratified by gender and age group (< 50 versus ≥ 50 years) were summarized. A 2-sided Wilcoxon rank sum test was used to compare the frequency of AD visits PPPY by treatment group.

A total of 589 C1-INH-HAE patients were identified from the claims database; the majority were female (69%) and 38% aged ≥ 50 years. A total of 313 patients (860 patient years) received pdC1-INH and 276 patients (729 patient years) “Other (non-C1-INH) treatments”. Overall, 76 patients (12.9%) had at least 1 visit for a coexisting AD. Based on coded diagnoses, the most common coexisting ADs were: lupus erythematosus (19 patients [3.2%]), alopecia (13 [2.2%]), rheumatoid arthritis, sicca syndrome, and connective tissue disorders (each 12 patients [2.0%]) (Table 1).

**Table 1:** Autoimmune conditions present in at least 1 visit

AI condition	Number (%) of patients		
	HAE index medication		Total (N = 589)
	Cinryze/Berinert	Firazyr/Kalbitor	
Any AI condition	42 (7.13)	34 (5.77)	76 (12.9)
Lupus erythematosus	12 (2.04)	7 (1.19)	19 (3.23)
Alopecia	6 (1.02)	7 (1.19)	13 (2.21)
Rheumatoid arthritis	9 (1.53)	3 (0.51)	12 (2.04)
Sicca syndrome	6 (1.02)	6 (1.02)	12 (2.04)
Connective tissue disorders	5 (0.85)	7 (1.19)	12 (2.04)
Crohn disease	3 (0.51)	6 (1.02)	9 (1.53)
Celiac disease	6 (1.02)	2 (0.34)	8 (1.36)
Raynaud’s disease	4 (0.68)	4 (0.68)	8 (1.36)
Thyroiditis	3 (0.51)	3 (0.51)	6 (1.02)
Psoriasis	2 (0.34)	4 (0.68)	6 (1.02)
Antiphospholipid syndrome	3 (0.51)	2 (0.34)	5 (0.85)
Rheumatism	2 (0.34)	–	2 (0.34)
Systemic sclerosis (scleroderma)	–	2 (0.34)	2 (0.34)
Ulcerative colitis	–	2 (0.34)	2 (0.34)
Nephritic syndrome	1 (0.17)	–	1 (0.17)

*AI* autoimmune, *HAE* hereditary angioedema, *N* total number of patients with HAE due to C1-inhibitor deficiency

The mean (95% CI) number of visits for ADs PPPY was lower in the pdC1-INH treatment (1.4 [0.562, 2.185]) than the “Other (non-C1-INH) treatments” (2.3 [0.832, 3.727]). Regardless of the treatment and age groups (< 50 and ≥ 50 years), the mean (95% CI) number of visits with ADs PPPY was higher in females than males. In the age group < 50 years, the mean (95% CI) number of AD visits with autoimmune diagnoses PPPY in pdC1-INH groups was 2.0 (0.381, 3.583) in females versus 0 in males, and 4.7 (1.178, 8.243) in females versus 0.6 [− 0.213, 1.505] in males for “Other (non-C1-INH) treatments” (Table 2).

**Table 2:** Mean visits per patient per year—any autoimmune condition and lupus erythematosus

Any autoimmune condition	Medication cohort	Number of patients^a^	Total f/u years	Number of visits	Autoimmune visits	Autoimmune visits per patient per f/u year				
						Mean (SD)	95% CI LB	95% CI UB	Range	p-value^b^
All	Other non-C1-INH	276	729	62,864	1528	2.3 (12.22)	0.832	3.727	0–107.03	0.7369
	pdC1-INH	313	860	85,859	1381	1.4 (7.30)	0.562	2.185	0–107.08	
< 50 years										
Male	Other non-C1-INH	56	136	6087	66	0.6 (3.21)	− 0.213	1.505	0–21.97	0.0733
	pdC1-INH	45	120	8082	0	0.0 (0.00)	0.000	0.000	0.0000	
Female	Other non-C1-INH	110	286	29,465	1188	4.7 (18.69)	1.178	8.243	0–107.03	0.6410
	pdC1-INH	146	386	44,613	1004	2.0 (9.79)	0.381	3.583	0–107.08	
Lupus										
All	Other non-C1-INH	276	729	62,864	324	0.6 (6.92)	− 0.189	1.451	0–105.23	0.375
	pdC1-INH	313	860	85,885	357	0.4 (2.65)	0.078	0.667	0–26.14	
< 50 years										
Male	Other non-C1-INH	56	136	6087	0	0.0 (0.00)	0.000	0.000	0–0	1.0000
	pdC1-INH	45	120	8082	0	0.0 (0.00)	0.000	0.000	0–0	
Female	Other non-C1-INH	110	286	29,465	315	1.6 (10.92)	− 0.499	3.629	0–105.23	0.6040
	pdC1-INH	146	386	44,613	236	0.5 (2.68)	0.025	0.900	0–25.91	

pdC1-INH treatment = Cinryze and Berinert and “Other non-C1-INH treatment” = Firazyr and Kalbitor

*C1*-*INH* C1-inhibitor, *CI* confidence interval, *f/u* follow-up, *LB* lower bound, *pd* plasma-derived, *SD* standard deviation, *UB* upper bound

^a^14 patients were missing age (8 females and 6 males)

^b^Two-sided Wilcoxon rank sum test

C1-INH-HAE may be linked with increased autoimmunity due to consumption of early components of the classical complement pathway and may be analogous to the increase in AD seen in patients with genetic deficiencies of C1 or C2 [4].

The estimated prevalence of ADs is 4.5% in the general population (2.7% in males and 6.4% in females) [5]. Only a few studies have been conducted which evaluated the association of C1-INH-HAE with ADs. In 1986, Brickman et al. systematically evaluated 157 patients with C1-INH-HAE and found that 19 patients (12%) had manifestations of ADs [6]. In 2011, Farkas et al. assessed the prevalence of ADs among 130 C1-INH-HAE and found a prevalence of 11.5% and also an increased severity of angioedema attacks in those with ADs [7].

In this present analysis, the prevalence of AD-related visits among 313 C1-INH-HAE patients treated with pdC1-INH was 13.4%, and prevalence of AD-related visits among 276 C1-INH-HAE patients treated with other (non-C1-INH) treatments was 12.3%. There is only a small difference in the prevalence of AD-related visits between these groups but still consistent with the benefit of the pdC1-INH treatment for reducing AD visits PPPY in C1-INH-HAE patients. Our analysis suggests a hypothesis that pdC1-INH replacement therapy may have a modulating effect on the occurrence and/or severity of ADs in C1-INH-HAE patients, possibly by increasing C1, C4 and/or C2.

This analysis has several limitations. Retrospective analyses of observational claims data over a limited period of time may be prone to unobserved confounders which can bias the observed associations. Therefore, these results can only be considered as hypothesis generating. The higher AD visit frequency observed for patients using treatments other than pdC1-INH for HAE may be associated with unobserved confounders, such as more severe HAE, or other factors that influenced their treatment decisions. Since claims data are based on professional coding for reimbursement purposes, some diagnoses may be missed, disregarded, or inaccurately applied. The purpose of the visits and reasons for the use of AD codes could not be verified in this study. The study also did not look at mode of treatment for ADs or number of frequency of treatments.

## Conclusion

Data from this claims database review showed that the C1-INH-HAE patients treated with pdC1-INH replacement therapy have a lower number of visits for coexisting ADs compared to those C1-INH-HAE patients treated with other methods. This provides a potential role of pdC1-INH replacement therapy in reducing or ameliorating the occurrence of AD in patients with C1-INH-HAE. Further research is needed to better understand the impact of pdC1-INH replacement therapy in modulating the severity and risk of concomitant AD in patients with C1-INH-HAE.

## Data Availability

All data are contained in the paper.

## Abbreviations

AD: Autoimmune disease
C1-INH: C1-esterase inhibitor
C1-INH-HAE: HAE due to C1-INH deficiency
CI: Confidence interval
HAE: Hereditary angioedema
ICD: International Classification of Diseases
pdC1-INH: Plasma-derived C1-INH
PPPY: Per patient per year
SD: Standard deviation
